# Geriatric Syndromes and Their Relationship with Mortality in a Population of Mexican Older Adults Aged 65 and Over, Admitted to the Emergency Department of a Second-Level Care Hospital

**DOI:** 10.3390/healthcare12121166

**Published:** 2024-06-08

**Authors:** José Juan Gómez-Ramos, Melissa González-Guerra, Ingrid Patricia Dávalos-Rodríguez, María Eloísa Pérez-Ruíz, Emiliano Peña-Durán, Alejandro Marín-Medina

**Affiliations:** 1 Especialidad de Medicina de Urgencias Adscrita al Centro Universitario de Ciencias de la Salud (CUCS), Universidad de Guadalajara, Guadalajara 44340, Mexico; josejuan79@yahoo.com (J.J.G.-R.); mely300194@hotmail.com (M.G.-G.); elo_0293@hotmail.com (M.E.P.-R.); 2 Departamento de Urgencias, Hospital General de Zona 89, Instituto Mexicano del Seguro Social (IMSS), Guadalajara 44100, Mexico; 3 Departamento de Biología Molecular y Genómicas, Centro Universitario de Ciencias de la Salud (CUCS), Universidad de Guadalajara, Guadalajara 44340, Mexico; ingriddavalos@hotmail.com; 4 Centro de Investigación Biomédica de Occidente (CIBO), Instituto Mexicano del Seguro Social (IMSS), Guadalajara 44340, Mexico; 5 Licenciatura en Médico Cirujano y Partero, Centro Universitario de Ciencias de la Salud (CUCS), Universidad de Guadalajara, Guadalajara 44340, Mexico; emilianodupe@live.com

**Keywords:** geriatric assessment, emergency service, hospital, dementia, frailty, delirium, aged

## Abstract

The main objective of this study was to analyze the relationship between Geriatric Syndromes (GSs) and in-hospital mortality in adults aged 65 and older admitted to the Emergency Department (ED). The study included 202 Older Adults (OAs) who met the inclusion criteria. We conducted a Comprehensive Geriatric Assessment and collected clinical and demographic data. A univariate analysis was carried out for each of the GSs analyzed. Those variables with *p* < 0.05 were entered into a multiple logistic regression using the backward stepwise entry method to analyze the independent predictor variables. The average number of GSs per individual was 4.65 (±2.76). Frailty syndrome was the most prevalent (70.2% of patients). Our study found an association between mortality and some GSs, such as frailty (*p* = 0.042), risk of falls (*p* = 0.010), delirium, cognitive impairment, dependence, and risk of ulcers (*p* < 0.001). We found that cognitive impairment (adjusted OR, 6.88; 95% CI, 1.41–33.5; *p* = 0.017) and dependence (adjusted OR, 7.52; 95% CI, 1.95–29.98; *p* = 0.003) were independent predictors associated with mortality in our population. It is necessary to develop new care strategies in the ED that respond to the needs of aging societies, including the use of new technologies and personnel with experience in gerontology.

## 1. Introduction

The population of individuals aged 80 and older is expected to triple in the next three decades, reaching 426 million [1]. The Americas region is not immune to these demographic changes; in a short time, it will transition from a young society to one consisting of many OAs [2].

GSs are “multifactorial health conditions that occur when the cumulative effects of deficiencies in multiple systems make an older person vulnerable to situational challenges” [3,4]. These syndromes are highly prevalent and adversely affect the quality of life, disability, and mortality of OAs, yet they remain undetected [5,6]. The incidence and prevalence of GSs vary according to the care setting, including the community, medical offices, and hospitals [7]. Even within the hospital, these figures differ depending on the specific care setting.

Overall, OAs represent 12% to 24% of all ED visits; they typically present a higher level of urgency and illness severity, more frequently arrive by ambulance, utilize more diagnostic testing, and have extended stays [8]. Additionally, they have a 2.5 to 4.6 times greater risk of hospitalization and a five times higher admission rate to an intensive care unit (ICU). OAs are also more likely to be misdiagnosed, leading to frequent discharge with unrecognized and untreated health problems [9].

EDs provide a critically important service to OAs by serving as an entry point for acute illnesses and providing continuous primary medical care 24 h a day. As the number of OAs seeking emergency care increases, it is essential to pay greater attention to utilization patterns, special care needs, and the effectiveness of current ED service delivery models for this population. Emergency physicians report more significant challenges in managing older patients with various clinical emergencies. We attribute these difficulties to factors such as atypical disease presentation, polypharmacy, and multiple comorbidities, which complicate the diagnosis and treatment of these patients [8,10].

In Mexico, there are no estimates of the incidence of GSs in OAs and it is unknown how these syndromes interact with each other as determinants of morbidity and mortality. OAs are known to have different care needs, as current emergency medical care models are not designed to adequately respond to the complex needs of OAs.

To our knowledge, this is the first study to determine the prevalence of GSs in Mexican OAs in the ED and determine its relationship with mortality. 

## 2. Materials and Methods

### 2.1. Study Population

This study was conducted at the ED of General Zone Hospital 89 of the Mexican Social Security Institute, in Guadalajara, Mexico; this hospital is a secondary-level medical center serving an approximate population of 530,000 inhabitants, with a global aging index of 55.8. Based on 94,920 people aged 65 years and older assigned to the Decentralized Management Medical Area No. 1042, zone 11 “Chapultepec”, the sample size was calculated, using the formula to estimate a finite population proportion [11,12], with a confidence level of 95% and a precision of 3%. The minimum sample size was estimated to be 202 patients. 

Patients were enrolled from October to November 2023, and only the first visit to the ED during the recruitment period was considered. We included patients with the following characteristics: (1) hospitalized in the general observation area of the hospital’s ED for any reason and with less than 12 h of stay; (2) aged 65 years or older; (3) capable of responding to the survey and signing an informed consent form, or having a responsible primary caregiver willing to accept the patient’s participation in the study, sign the informed consent form on their behalf, and provide information about the patient’s health status. We excluded patients with the following characteristics: (1) patients under mechanical ventilation, (2) patients who were not part of the hospital’s enrolled population, and (3) patients who were transferred to another hospital for definitive treatment continuation, making it impossible to verify the outcome of their hospitalization.

### 2.2. Data Collection

All patients included in the study, or their primary caregivers if applicable, underwent a sociodemographic survey that included data such as age, educational level, employment status, family composition, income, and its source. Additionally, other general clinical aspects were collected, such as conditions at the time of hospital arrival, companions at the time of arrival, alcohol and tobacco consumption habits, presence of comorbidities, patterns of emergency service utilization, geriatric care service utilization, and length of hospital stay. 

#### Geriatric Assessment

The clinical assessment included recording some physiological variables, while the comprehensive GAs included: (1) Charlson Comorbidity Index (CCI); (2) polypharmacy (defined as consumption of ≥5 medications at the time of evaluation; although, inappropriate prescribing was also evaluated according to the 2019 Beers Criteria); (3) cognitive impairment (defined by the presence of ≥3 errors on the Six-Item Screener, SIS); (4) affective disorders (defined as the presence of ≥5 of the 9 depression criteria, present on at least half of the days in the past two weeks, and if one of the symptoms is depression or anhedonia, according to the Patient Health Questionnaire-9, PHQ-9); (5) frailty (considered frail, with a score of ≥5 according to the Clinical Frailty Scale, CFS); (6) dependence (defined as a score of ≤4 on the modified Katz index that evaluates Activities of Daily Living, ADLs); (7) risk of falls (defined as a score ≥ 3 on the Downton Fall Risk Index, DFRI); (8) urinary incontinence (defined as any score > 0 on the International Consultation on Incontinence Questionnaire, Short Form Spanish, Chile, ICIQ-SF-SC); (9) delirium (defined according to the Confusion Assessment Method as the presence of criteria 1, 2, and 3 or 4, CAM); and (10) risk of pressure ulcers (defined by a score of ≤14 points on the Braden Scale, BS). In addition to the GAs, nutritional evaluation was performed by calculating each participant’s Body Mass Index (BMI kg/m^2^) using the Quetelet formula [13], using the calculated weight and height formulas, useful in the acute clinical setting with patients with disabling pathologies or those who are bedridden; in the case of weight, the general formula was used, and in the case of height, the formula validated in the Mexican American population was used [14]. The Glomerular Filtration Rate (GFR) was calculated using the formula proposed by Levey et al. [15], adjusted for race and sex. The main outcome was in-hospital mortality for any cause after admission to the ED, which was obtained through the electronic health record.

### 2.3. Statistical Analysis

Exploratory analysis of the data was carried out using descriptive measures. We presented all continuous variables as means ± standard deviations and expressed categorical variables as percentages. For continuous variables, the non-parametric Kolmogorov–Smirnov test was performed to evaluate the normal distribution of the data. Accordingly, the Mann–Whitney U test was used for comparison between groups. For qualitative variables, Pearson’s chi-squared test and Fisher’s exact test were used as appropriate. Finally, all those variables with a *p*-value of less than 0.05 were entered into the multiple logistic regression using the backward stepwise entry method to analyze the independent predictor variables. Multicollinearity among all the covariates was evaluated using the Variance Inflation Factor (VIF). A VIF > 10 indicates serious multicollinearity, while a VIF value > 5 was considered indicative of a high correlation of the variables [16]. We conducted all statistical analyses using the Statistical Package for the Social Sciences (IBM^®^ SPSS^®^ Statistics) for Windows, version 24.0 (IBM Corp., Armonk, NY, USA). We considered a *p*-value < 0.05 as statistically significant.

### 2.4. Ethical Considerations

The study adhered to the Regulations of the General Health Law on Research in Mexico (Title II, ‘Ethical Aspects of Research in Human Beings’, Chapter 1, Articles 13 to 27) and the principles of the Declaration of Helsinki.

The Local Ethics and Health Research Committee of General Zone Hospital 89 (1307) reviewed and approved the study protocol, with approval number R-2023-1307-101. Only individuals who presented for the first time to the ED during the recruitment period were considered eligible to participate in the study. All patients or their legal representatives provided signed informed consent.

## 3. Results

### 3.1. Sociodemographic Characteristics and Clinical History of the Patients

We enrolled a total of 202 OAs aged >65 years who were in the observation room of the ED from October to November 2023. Table 1 shows the distribution by age, sex, and other sociodemographic characteristics of the OAs included in the study. Forty-eight percent were women.

When analyzing personal pathological history (Table 2), a high prevalence of diabetes mellitus (DM), as well as moderate-to-severe chronic kidney disease, and cardiovascular disorders (represented by peripheral vascular disease, acute myocardial infarction, and cerebrovascular events) was observed. 

Upon admission to the ED, each patient’s clinical status was generally good (Table 3); most had a good nutritional status (BMI = 19.7). We observed that glucose levels and levels of nitrogenous waste were elevated.

### 3.2. Geriatric Assessment

Our study found an association between mortality and some GSs, such as frailty (*p* = 0.042), risk of falls (*p* = 0.010), delirium, cognitive impairment, dependence, and risk of ulcers (*p* < 0.001) (Table 4). Regarding the GAs, we observed that only 20% of the patients had received a geriatric evaluation in their lifetime, and many of these evaluations occurred during a previous hospitalization.

Hospitalized OAs showed some degree of functional impairment in 30% of cases. Dependency for activities of daily living ranged from moderate to severe in 32.1% of cases according to the KATZ index, predominantly of type G (dependent in all activities), followed by type H (dependent in two activities but not classified in C, D, E, and F).

Upon admission, 70% of OAs exhibited varying degrees of frailty, with mild frailty predominating, and a decrease in frequency as frailty increased; linked to immobility, the risk of ulcers was present in 39.1% of hospitalized OAs, with a high risk according to the Braden Scale.

Additionally, 39.1% of OAs had urinary incontinence upon admission, predominantly stress incontinence, and 57.4% presented with dementia syndrome; this contrasted with only 5% of patients being known to have dementia or diagnosed with dementia upon admission. In comparison, 25.7% of OAs had delirium, most likely related to some acute complication upon admission to the ED.

Two of the most representative syndromes were affective disorder in 60.3% and falls syndrome in 69.8%, while 43% of OAs presented with malnutrition, predominantly being underweight.

Regarding polypharmacy syndrome, one of the syndromes with the most significant conceptual difficulties, the mean number of medications taken by OAs was 3.7, with 34.15% of OAs taking five or more medications; 51% took inappropriate medications according to the 2019 Beers Criteria, with hormonal medications and NSAIDs being the most consumed. Although some patients were observed to take inappropriate medications according to their GFR, as well as medications with a strong cholinergic effect and medications that interact with each other, the prevalence was low (5.9%, 2.47%, and 0.9%, respectively).

### 3.3. Geriatric Syndromes and Their Relationship with Mortality

Finally, fall risk (DFRI), frailty (CFS), dependence (ADL), risk of ulcers (BS), cognitive impairment (SIS), delirium (CAM), Charlson index, and the presence of previous GAs (all with a *p*-value < 0.05), were entered into a binomial logistic regression model using backward stepwise entry. The independent predictors of mortality for OAs admitted to the ED were cognitive impairment (SIS) (adjusted odds ratio [OR], 6.88; 95% confidence interval [CI], 1.41–33.5; *p* = 0.017) and dependence (ADL) (adjusted OR, 7.52; 95% CI, 1.95–29.98; *p* = 0.003) (Table 5). We assessed the covariates’ multicollinearity using the VIF in this analysis, and all the VIFs were between 1.0 and 3.0.

## 4. Discussion

The specific objectives of this study were to identify the clinical and sociodemographic characteristics of OAs admitted to a second-level emergency service in Mexico, determine the prevalence of the main GSs in our study population through GAs, and analyze the relationship between GSs and in-hospital mortality for any cause once admitted to the ED. 

In our study, we found that the three most common GSs were frailty (70.2%), instability and falls (60.3%), and dementia (57.4%), with an average of 4.65 (±2.76) syndromes per individual; therefore, the coexistence of GSs is common to a greater or lesser extent. In Mexico, through the National Health and Nutrition Survey 2018–2019 (ENSANUT 2018–2019), a prevalence of frailty of 10.6% was determined (23.0% being pre-frail). Moreover, depressive symptoms were determined in 40.6%, and visual and auditory impairment in 13.1% and 6.9%, respectively. In addition, 5.6% presented with cognitive disorders and 19.6% presented with mobility disorders. It is also important to mention that 4.2% had difficulties with activities of daily living. As can be seen, there are significant differences with our findings; since the ENSANUT survey involves only self-reporting, is not carried out by trained personnel, and is conducted at the community level; however, it shows that some GSs coexist in the community to a greater or lesser extent, and that in the presence of a triggering stimulus such as the exacerbation of a pre-existing or de novo pathological state, they often lead to adverse results [17].

In our study, frailty emerged as the most frequent syndrome, with results very similar to those in other countries [18], confirming that frailty is one of the main predictors of hospitalization in emergency services for OAs living in the community [19]. Following frailty, the falls syndrome (represented in our study by the existing risk of falls on hospital admission) was highly prevalent in our population, contrasting with findings reported in other studies, which report a low prevalence of 14% that increases by 31% after the first fall [20]. Our study found cognitive impairment to be the third most common GS. The prevalence of cognitive impairment in our population was like that reported in other populations [21,22,23,24]. This syndrome has gone unnoticed in some cases at the community level, so it is necessary to carry out intentional screening for cognitive impairment in OAs both upon admission to the ED and at the community level. This allows for the development of interventions focused on primary care and recovery strategies for prevention in the community. A notable case is acute confusional syndrome or delirium, which in our population was found to have a high prevalence of compared to other populations [25,26,27] and to that observed in specialized geriatric care centers [28].

In the Mexican population of OAs that we studied, the prevalence of dependence in terms of activities of daily living measured through the Katz Index was like that reported in other studies [29,30,31,32,33]. It is known that during an acute health problem, OAs can become dependent, increase their level of dependency, or even lose their functional independence. Additionally, it has been observed that dependency is associated with increased use of emergency services, a more significant burden for caregivers [29], and a strong bond with other GSs [31,32], which collectively contributes to a higher risk of adverse health outcomes, including increased short- and long-term mortality, even after the patient has been discharged. In relation to pressure ulcers, it has been observed that this is a parameter that can be used as an early predictor of in-hospital mortality [34].

It is essential to mention that in our population, only 20% of OAs had received a geriatric evaluation in their lifetime, the majority in the context of a previous hospitalization. Therefore, there are a low percentage of OAs in Mexico who access geriatric care services, which is associated with higher mortality. This contrasts with intervention models in other countries using strategies such as telemedicine or implementing the use of health personnel with gerontology experience in emergency services [35,36]. GAs in the ED are often brief, as ED care models are mostly unidimensional and focus on the reason for hospital admission [37,38]. Therefore, it is imperative to develop models of care for OAs in primary health care services that include prior GAs, not only in the context of hospitalization but also in terms of the availability of personnel with gerontological experience in emergency services, as this could significantly impact the morbidity and mortality of OAs. 

Our study found an association between mortality and some GSs, such as frailty, dependence, delirium, risk of ulcers, cognitive impairment, and risk of falls; it is essential to mention that all these syndromes have a multifactorial etiological origin and are interrelated [39].

When analyzing the correlation between the factors observed in OAs admitted to the ED and in-hospital mortality (Table 5), in our study it was found that the independent predictors of mortality for OAs admitted to the ED were cognitive impairment (SIS) (adjusted odds ratio [OR], 6.88; 95% confidence interval [CI], 1.41–33.5; *p* = 0.017) and dependence (ADL) (adjusted OR, 7.52; 95% CI, 1.95–29.98; *p* = 0.003). Few studies have investigated the relationship between GSs and mortality, particularly in the ED. In a study conducted in a Chinese population, multimorbidity, incontinence, and activities of daily living were found to be independent predictors of mortality at six months [8]. We can attribute the differences between the independent predictor variables to the models of care and evaluation of OAs used in emergency services and primary medical care, as well as to the characteristics of each population. Therefore, we must develop strategies focused on multidisciplinary evaluations, incorporating new technologies into the medical care of OAs, and increasing routine geriatric evaluations. These efforts aim to reduce hospitalizations and emergency room saturation, thereby improving health and the quality of care for patients and effectively responding to the needs of aging societies.

Our study had some limitations, including a small sample size and the evaluation of patients from only a single hospital center in a specific region of the country. Additionally, the difficulties previously described in calculating somatometry in frail, bedridden, and hospitalized patients under acute conditions required us to use formulas for calculation. It is necessary to conduct multicenter studies with a larger sample size to obtain a broader context of GAs in OAs in EDs in Mexico.

## 5. Conclusions

GSs of frailty, delirium, and instability, along with comorbidities such as DM and neoplasia, are associated with an increased risk of mortality. Non-contributory pensions represent a large percentage of the income of the elderly population. We found an average of 4.65 (±2.76) GSs per individual. Only 20% of our population had received a prior geriatric evaluation, mainly in the context of hospitalization. We found cognitive impairment and dependency to be independent predictors of mortality in our population. There is an urgent need to develop intervention models based on health education and prevention in the community to improve the quality of life of OAs in Mexico.

## Figures and Tables

**Table 1:** Sociodemographic characteristics of OAs hospitalized in the ED.

Variable		Values
Age		75.76 (±7.71)
Sex	Men	105 (52.00)
	Women	97 (48.00)
Marital Status	Married	101 (50.00)
	Single	85 (42.10)
	Widowed	13 (6.40)
	Common-law marriage	3 (1.50)
Employment status	Retired	117 (57.90)
	Household	49 (24.30)
	Unemployed	23 (11.40)
	Employed	13 (6.40)
Family nucleus	Nuclear family	108 (53.50)
	Children’s home address	68 (33.70)
	Lives alone	22 (10.90)
	Retirement home	4 (2.00)
Hospital arrival	Own motor vehicle	122 (60.40)
	Ambulance	49 (24.30)
	Regulated fare transportation	28 (13.90)
	Public transportation network	2 (1.00)
	Walking	1 (0.50)
Companion	Son/daughter	127 (62.90)
	Spouse	47 (23.30)
	Other family members	20 (9.90)
	Other non-family person	8 (4.00)
Source of income	Own work	13 (6.40)
	Non-contributory pensions	188 (93.10)

Note. Categorical variables are expressed as frequency (n) and percentage (%). Continuous variables are expressed as means and standard deviations (±SD).

**Table 2:** Pathological history of OAs hospitalized in the ED.

Comorbidities	Values	*p*-Value
Diabetes Mellitus	92 (45.50)	**0.016** ^b^
Moderate/Severe Chronic Kidney Disease	79 (39.10)	0.570 ^a^
Benign Prostatic Hyperplasia	30 (14.90)	0.320 ^b^
Chronic Obstructive Pulmonary Disease	29 (14.40)	0.176 ^b^
Cerebrovascular Event	25 (12.40)	0.720 ^b^
Acute Myocardial Infarction	21 (10.40)	1.000 ^b^
Neoplasms	19 (9.40)	**0.027** ^b^
Peripheral Vascular Disease	17 (8.40)	0.385 ^b^
Congestive Heart Failure	13 (6.40)	0.371 ^b^
Ulcerative Disease	11 (5.40)	0.298 ^b^
Chronic Hepatopathy	11 (5.50)	1.000 ^b^
Dementia	10 (5.00)	0.064 ^b^
Connective Tissue Disease	5 (2.50)	1.000 ^b^
Hemiplegia	3 (1.50)	1.000 ^b^
Tobacco consumption	57 (28.20)	0.172 **^a^**
Harmful alcohol consumption	182 (90.10)	0.825 **^a^**

Note. Univariate analysis. Categorical variables are expressed as frequency (n) and percentage (%). ^a^ Pearson’s chi-squared test. ^b^ Fisher’s exact test. Statistically significant values in bold (*p* < 0.05).

**Table 3:** Upon admission to the ED, physical status and physiological and biochemical constants of OAs.

Variable		Values
Nutritional assessment	Calculated height (m)	1.60 (±9.00)
	Calculated weight (kg)	51.48 (±13.00)
	Calculated BMI (kg/m^2^/BSA)	19.75 (±4.10)
	Calculated Glomerular Filtration Rate (GFR) (mL/min)	68.29 (±44.27)
Nutritional status	<18.5 (underweight)	73 (36.10)
	18.5–24.9 (normal weight)	114 (56.40)
	25.0–29.9 (overweight)	12 (5.90)
	>30.0 (obese)	3 (1.50)
Physiological constants	Systolic blood pressure (mmHg)	122.04 (±27.70)
	Diastolic blood pressure (mmHg)	69.35 (±14.16)
	Heart rate (bpm)	85.61 (±19.44)
	Respiratory rate (rpm)	20.1 (±3.33)
	Temperature (°C)	36.29 (±0.42)
Laboratory results	Central glucose (mg/dL)	131.26 (±97.82)
	Creatinine (mg/dL)	1.94 (±2.37)
	Urea (mg/dL)	73.88 (±66.27)

Note. We calculated percentages for categorical variables, and means and standard deviations for quantitative variables. Abbreviations: m, meters; kg, kilograms; kg/m^2^/BSA, kilograms per square meter of body surface area; mL, milliliters; min, minute; mmHg, millimeters of mercury; bpm, beats per minute; rpm, respirations per minute; °C, degrees Celsius; mg/dL, milligrams per deciliter.

**Table 4:** Geriatric assessment of OAs hospitalized in the ED on admission and its association with in-hospital mortality.

Variable	Values	*p*-Value
**Previous geriatric assessment**	43 (21.3)	**0.017 ^b^**
**Charlson Index**	5.55 (±2.00)	**0.002 ^c^**
**Hospitalizations**		
Hospitalization in the previous month	43 (21.30)	0.386 ^b^
Number of hospitalizations in the previous month	0.23 (±0.49)	0.294 ^c^
**Geriatric Syndromes**		
Frailty	142 (70.20)	**0.042** ^a^
Dependence	65 (32.10)	**<0.001** ^a^
Risk of ulcers	79 (39.10)	**<0.001** ^a^
Urinary incontinence	116 (57.40)	0.094 ^a^
Cognitive impairment	83 (41.00)	**<0.001** ^a^
Delirium	52 (25.70)	**<0.001** ^a^
Affective disorders (depression)	122 (60.30)	0.355 ^a^
Risk of falls	141 (69.80)	**0.010** ^a^
Polypharmacy		
More than four medications	69 (34.15)	0.562 ^a^
Inappropriate medications	104 (51.40)	0.899 ^a^
Hormonal	28 (13.80)	1.000 ^b^
Nonsteroidal anti-inflammatory drugs	25 (12.30)	0.720 ^b^
Benzodiazepines	22 (10.80)	1.000 ^b^
Calcium antagonists	16 (7.92)	0.663 ^b^
Antithrombotics	15 (7.40)	1.000 ^b^
Oral hypoglycemic agents	10 (4.90)	0.603 ^b^
Consumption of medications with strong cholinergic effects	5 (2.47)	0.410 ^b^
Drug interaction	2 (0.90)	1.000 ^b^
Inadequate according to GFR	12 (5.90)	0.102 ^b^
Average number of medications consumed	3.71(2.54)	0.563 ^c^
Number of inappropriate medications	0.83 (1.01)	0.692 ^c^

Note. Univariate analysis. Categorical variables are expressed as frequency (n) and percentage (%). ^a^ Pearson’s chi-squared test. ^b^ Fisher’s exact test. ^c^ Mann–Whitney U test. Statistically significant values in bold (*p* < 0.05). GFR, glomerular filtrate rate.

**Table 5:** Multiple logistic regression analysis of independent predictors and odds ratio of in-hospital mortality among patients hospitalized in the ED.

Independent Simple Pearson’s Chi-Square Test		Multiple Logistic Regression		
Variable	*p*-Value	OR	95% CI of OR	*p*-Value
Cognitive impairment, SIS	<0.001	6.88	[1.41–33.5]	0.017
Dependence, ADLs	<0.001	7.52	[1.95–28.98]	0.003

Note. The independent variables with a *p*-value < 0.05 included in the model were: fall risk, frailty, dependence, risk of ulcers, cognitive impairment, delirium, Charlson index, and the presence of previous geriatric assessment. SIS, Six-Item Screener; ADLs, Activities of Daily Living.

## Data Availability

The data presented in this study are available upon request from the corresponding author due to our institution’s policies.
